# Preemptive oxycodone is superior to equal dose of sufentanil to reduce visceral pain and inflammatory markers after surgery: a randomized controlled trail

**DOI:** 10.1186/s12871-019-0775-x

**Published:** 2019-06-11

**Authors:** Yi An, Lei Zhao, Tianlong Wang, Jiapeng Huang, Wei Xiao, Ping Wang, Lixia Li, Zhongjia Li, Xiaoxu Chen

**Affiliations:** 1 Department of Anesthesiology, Xuanwu Hospital, Capital Medical University, 45 Changchun Street, Xicheng District, Beijing, 100053 China; 2 National Clinical Research Center for Geriatric Disorders, Beijing, China; 3 Department of Anesthesiology, Jewish Hospital and Department of Anesthesiology & Perioperative Medicine, University of Louisville, Louisville, KY USA

**Keywords:** Preemptive analgesia, Oxycodone, Sufentanil, Visceral pain, TNF-α

## Abstract

**Background:**

Postoperative visceral pain is common after surgery and previous studies have demonstrated that oxycodone is an effective treatment. In this study, we compared the effects of preemptive oxycodone to equal dose of sufentanil on postoperative pain and serum level of inflammatory factors (TNF-α, IL-6, IL-10) after laparoscopic cholecystectomy.

**Methods:**

Forty patients undergoing laparoscopic cholecystectomy were randomized into preemptive oxycodone group or preemptive sufentanil group.

Patients were given either oxycodone 0.1 mg/kg (oxycodone group, *n* = 20) or sufentanil 0.1 μg/kg (sufentanil group, n = 20) for preemptive analgesia. We evaluated pain/sedation scores at 0 h, 0.5 h, 2 h, 4 h, 6 h, 8 h and 24 h after surgery and measured serum concentrations of TNF-α, IL-6 and IL-10 before surgery and at 0 h, 6 h and 24 h after surgery.

**Results:**

Twenty patients were recruited in each group. Numerical rating scale (NRS) of visceral pain in the oxycodone group at 2 h when resting (0.5(0,2.75) vs 3(2,4), *P* = 0.008) and moving (0.5(0,3) vs 3(2.25,4), *P* = 0.015) and 4 h when moving (2(0,3) vs 3(0,4.75), *P* = 0.043) after surgery were significantly lower than the sufentanil group. Serum concentrations of TNF-α at 6 h (38.68 ± 10.49 vs 73.02 ± 16.27, *P*<0.001) and 24 h (43.12 ± 8.40 vs 74.00 ± 21.30, *P*<0.001) in the oxycodone group were lower than the sufentanil group.

**Conclusions:**

Preemptive oxycodone 0.1 mg/kg administration could effectively suppress visceral pain at 2 h and 4 h after surgery and had lower inflammatory marker, serum TNF-α, level when compared to equal dose of sufentanil.

**Trial registration:**

Clinical trials registration number: ChiCTR-IOR-17013738http://www.chictr.org.cn/showproj.aspx?proj=17346. Date of registration: 6th December 2017.

## Background

Laparoscopic cholecystectomy (LC) carries short operation time, mild surgical trauma and minimal blood loss, which could greatly enhance recovery after surgery (ERAS) [1, 2]. However, acute pain after LC causes severe physical discomfort, prolong the recovery duration and delay discharge [3, 4]. LC postoperative pain consists of incision pain, visceral pain and referred pain (shoulder pain). Visceral pain is a major part of pain within 24 h after surgery [5] and is difficult to locate and lack targeted treatments. Organ injury and peritoneal inflammation, local acidosis and visceral mucosa ischemia caused by pneumoperitoneum may be causes of visceral pain after LC surgery [2, 6]. Moreover, Morten et al. [7] have shown that patients with higher visceral pain in the first week after LC were associated with chronic pain.

Degree of analgesia intraoperatively is another factor impacting postoperative pain. The nociceptive stimulation during operation will increase pro-inflammatory cytokines such as TNF-α, IL-6 and decrease anti-inflammatory cytokines such as IL-10 [8] and cause systemic inflammatory response, which will influence recovery process and prognosis.

Opioids are the most common medication used by anesthesiologists to treat pain. There are three opioids receptors, μ receptor, κ receptor and δ receptor. Sufentanil is high selective μ receptor agonist with fast onset and strong analgesic effect [9, 10]. However, sufentanil is less effective to relieve visceral pain than oxycodone [11]. Oxycodone not only activate the μ receptor but also occupy the κ receptor and relieve pain of organs composed of smooth muscle [12].

Besides drug types, the timing of drug delivery also impacts perioperative pain. Preemptive analgesia starts administration of analgesic interventions before nociceptive stimulation and is intended to suppress the progress of stress state and central sensitization before they start [13, 14]. Previous studies have shown a reduction in postoperative pain and use with preemptive analgesia [13, 15].

We hypothesized that preemptive oxycodone can reduce the degree of postoperative visceral pain and decrease the incidence of postoperative remedial analgesia and PONV. The primary outcome was to evaluate whether preemptive oxycodone administration is superior to equal dose of sufentail on visceral pain after LC in postoperative time points. Secondary outcomes included the serum level of TNF-α, IL-6 and IL-10, the dose of opioid used intraoperative, the postoperative sedation level and the degree of postoperative nausea and vomiting (PONV) and complications after surgery.

## Methods

This double-blinded prospective randomized controlled trial was conducted from March 2018 to September 2018 at the Xuanwu Hospital, Beijing, China, after approval from Xuanwu Hospital Ethics Committee in March 2018. The trial was registered with China Clinical Trial Registry (registration number: ChiCTR-IOR-17013738, principal investigator’s name: Lei Zhao, date of registration: 6th December 2017). Forty patients scheduled for LC were included and the written informed consents were obtained from all patients before participation. This manuscript adheres to the applicable CONSORT guidelines.

Inclusion criteria were patients aged 18–65 years, with an American Society of Anesthesiologists (ASA) physical status I-II, body mass index 18-35 kg/m^2^ [16]. Patients with severe systematic disease, history of gastrointestinal hemorrhage or peptic ulcer, suffering acute or chronic pain, dependence on alcohol or opioid, using psychological drugs, allergic to agents used in this trial were excluded. Patients who had to be converted to open surgery, used drugs out of initial anesthetic plan (inhalational anesthetic agents, other analgesics, et al.), experienced special complications (stroke, acute coronary syndrome, circulatory instability caused by arrythmia, liver injury, et al.) during surgery were excluded from this trial.

Forty patients were randomly assigned into either oxycodone group (group O, *n* = 20) or sufentanil group (group S, n = 20) using a computer-generated list by anesthesiologist who is not involved in this trial. Allocation was blinded to patients, anesthesiologists and other healthcare providers. Numerical rating scale (NRS) and characteristics of LC postoperative pain were explained to patients during preoperative interview. Incisional pain is defined as a pain restricted to incision site on abdominal wall, visceral pain is defined as a deep, dull and hard to locate pain, while the referred pain is defined as the right shoulder pain in LC. Patients were educated to distinguish these three types of pain from our definitions.

Heart rate (HR), blood pressure (BP), SpO_2_ were monitored after entering the operation room and Bispectral index (BIS) electrodes were placed on patients’ forehead. After adequate preoxygenation with 100% oxygen, a bolus of 0.1 mg/kg oxycodone was administered as preemptive analgesia [15, 17] in oxycodone group followed by sufentanil 0.2μg/kg, etomidate 0.2 mg/kg and rocuronium 0.6 mg/kg as anesthesia induction. Patients in sufentanil group were administered sufentanil 0.1 μg/kg as preemptive analgesia followed by sufentanil 0.2μg/kg, etomidate 0.2 mg/kg and rocuronium 0.6 mg/kg as anesthesia induction. Tracheal intubation was performed 5 min after preemptive oxycodone/sufentanil administration. Ventilation model was PCV-VG, tidal volume 6-8 ml/kg, FiO_2_ 50%, I: E = 1:2, and P_ET_CO_2_ was monitored and maintained between 35 and 45 mmHg. Anesthesia was maintained with total intravenous anesthesia (TIVA) using propofol and remifentanil by target controlled infusion (TCI) pumps after tracheal intubation was accomplished. Propofol was infused using Marsh model with 1μg/ml as initial concentration and adjusted by ±0.5μg/ml. Remifentanil was infused using Minto model with 4 ng/ml as initial concentration and adjusted by ±1 ng/ml. The target was to maintain BIS between 40 and 60 and have no body movements during the whole process of operation.

BP and HR were stabilized between ± 20% of baseline. Parecoxib 40 mg was given 30 min before the end of surgery in both groups. Ondansetron 4 mg and oxycodone 0.05 mg/kg were administered when pneumoperitoneum was stopped and local anesthesia was administrated with 0.5% ropivacaine.

Extubation was performed after patients met extubation criteria. All participants were immediately (0 h) investigated for degree of incision pain, visceral pain and shoulder pain (NRS) when resting (breathing) or moving (cough and deep breathing). Degree of sedation and PONV were also evaluated. In post anesthesia care unit (PACU), oxycodone 2 mg was administered as remedial anesthesia if NRS ≥ 4 and it could be repeated every 10 min until NRS ≤ 3. Researchers recorded the drug amount and pain assessments in PACU. Follow-up evaluations were conducted at 0.5 h, 2 h, 4 h, 6 h, 8 h and 24 h postoperatively by anesthesiologists blinded to grouping and offered the same questions to patients. Postoperative complications were also recorded.

Blood samples were collected before operation (T0), at the end of operation (T1), 6 h (T2) and 24 h (T3) after operation. Serum samples were stored at − 80 °C refrigerator after centrifugation with 3000 rpm for 15 min and used ELISA to measure serum level of TNF-α, IL-6 and IL-10.

### Statistical analysis

The result of our pilot experiment was used as reference standard of the calculation of sample size. Setting an α of 0.05(2-tailed) and a power of 80%, the sample size was 17 in each group calculated by the formula n = [Zα/2 + Zβ]2 /(P1-P2)^2^ and a sample size of 20 patients per group was chosen to allow for potential drop-outs. Data were analyzed by SPSS version 17.0. Continuous data were presented as mean and standard deviation (SD) and non-continuous data in median (quartile). The demographics and intraoperative situations were compared by Student *t* test or χ^2^ test. The NRS, degree of sedation and PONV were compared with Mann-Whitney U test. Serum concentration of TNF-α, IL-6 and IL-10 were analyzed by repeated measurement analysis of variance, *P* < 0.05 was considered as a statistical significance.

## Results

**Fig. 1:**
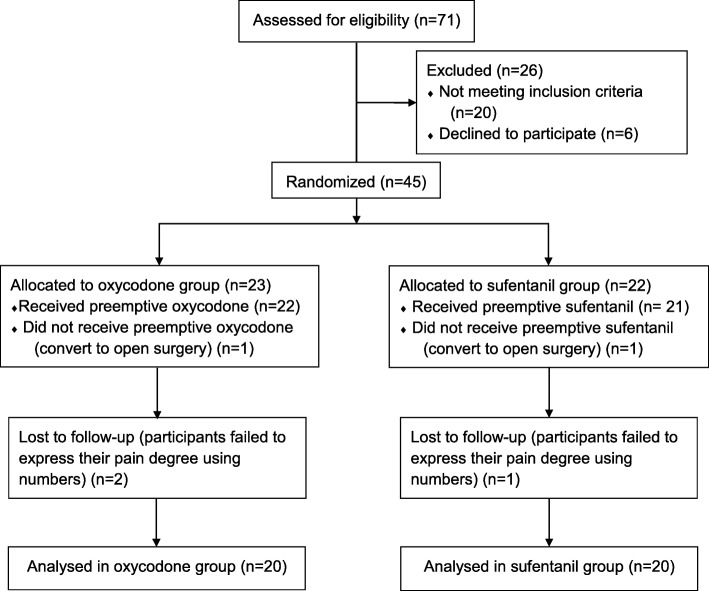
Flow diagram. ASA, American Society of Anesthesiologist

Seventy one patients were assessed for eligibility based on inclusion and exclusion criteria, with 26 patients excluded for not meeting inclusion criteria due to poor physical status (*n* = 10), emergency surgery (*n* = 6), long term psychotropic drugs use (*n* = 4) and refusing to participation (n = 6). After surgery, 5 patients were excluded for converting to open surgery and failing to follow up as planned. Finally, 40 patients were included in present analysis (group O: group S = 1:1, *n* = 20) (Fig. 1).

Demographics and intraoperative characteristics of all participants are summarized in Table 1 and no statistical significance was found between the groups.

**Table 1:** Demographic Data and Intraoperative Characteristics

	Group O (*n* = 20)	Group S (*n* = 20)	*P* value
Gender (male/female)	9/11	4/16	0.177
Age (year)	48 ± 13	51 ± 12	0.429
BMI (kg/m2)	25 ± 4	26 ± 4	0.718
ASA(I/II)	5/15	5/15	1.000
Cholecystolithiasis/gallbladder polyps	16/4	19/1	0.339
Pneumoperitoneum time (min)	36 ± 17	29 ± 9	0.201
Operation time (min)	51 ± 20	41 ± 11	0.149
Anesthesia time (min)	78 ± 21	68 ± 15	0.142
Wake-up time (min)	9 ± 3	8 ± 3	0.509
Extubating time (min)	12 ± 3	12 ± 4	0.964
Bile spillover(Y/N)	6/14	3/17	0.449
Blood loss (ml)	Minimal	Minimal	NA
Drainage(Y/N)	5/15	1/19	0.184

Data were expressed as mean ± SD

*ASA* American Society of Anesthesiologists, *BMI* body mass index, *Group O* oxycodone group, *Group S* sufentanil group, *Y* yes, *N* no

Medication quantities and perioperative complications are shown in Table 2 and no statistical significance was found between the groups. The incidence of nausea and vomiting in the oxycodone group is similar to the sufentanil group without statistical significance. Degree of sedation was evaluated using Ramsey scale and no statistical significance was observed between the groups.

**Table 2:** Medication Quantities and Complications

	Group O	Group S	*P* value
Propofol (mg/kg/h)	4.84 ± 0.92	5.07 ± 1.46	0.799
Remifentanil (μg/kg/min)	0.26 ± 0.07	0.27 ± 0.06	0.253
Hypertension (Y/N)	3/17	1/19	0.598
Hypotension (Y/N)	16/4	16/4	1.000
Tachycardia (Y/N)	1/19	2/18	1.000
Bradycardia (Y/N)	0/20	1/19	1.000
Myocutaneous antagonist (Y/N))	4/16	8/12	0.301
Remedial analgesia (Y/N)	3/17	5/15	0.693
Exhaust time (h)	15 ± 10	13 ± 7	0.858
PONV (Y/N)	17/3	17/3	1.000

Either nausea or vomiting is recorded as “Y”

*Group O* oxycodone group, *Group S* sufentanil group, *PONV* postoperative nausea and vomiting, *Y* yes, *N* no

**Fig. 2:**
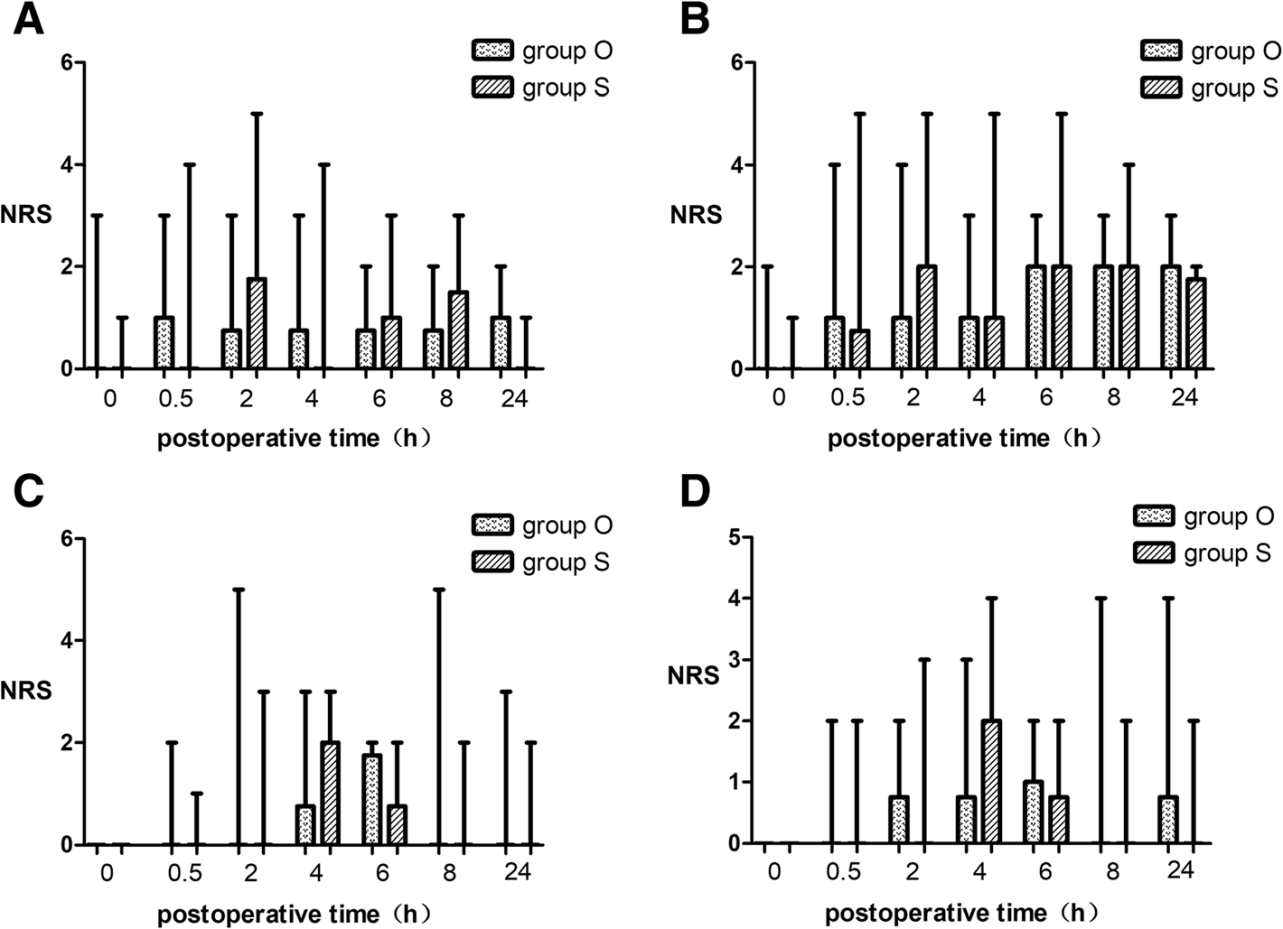
Incision Pain and Shoulder Pain when resting and moving. The results are represented by box graph. The transverse line in rectangular box is the median, the lower bound means the lower quartile and the upper bound is the upper quartile. The lower/upper bar is the minimum/maximum. **a**. incision pain when resting. **b**. incision pain when moving. **c**. shoulder pain when resting. **d**. shoulder pain when moving. No statistical significance was found. Group O = oxycodone group. Group S = sufentanil group

The incision pain, visceral pain and shoulder pain when resting or moving were followed at 0 h, 0.5 h, 2 h, 4 h, 6 h, 8 h, 24 h after surgery. Figure 2 shows the incision pain and shoulder pain when resting or moving and no statistical significance was found.

**Fig. 3:**
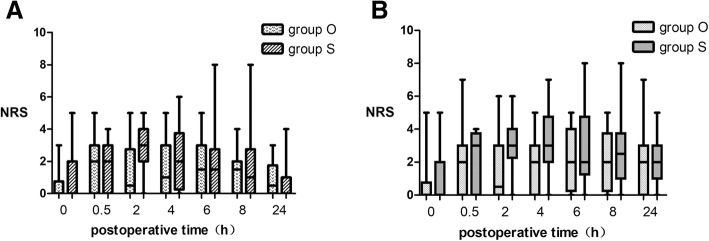
Visceral Pain when Resting and Moving. The results are represented by box graph. The transverse line in rectangular box is the median, the lower bound means the lower quartile and the upper bound is the upper quartile. The lower/upper bar is the minimum/maximum. **a**. Visceral pain when resting. **b**. Visceral pain when moving. Group O = oxycodone group. Group S = sufentanil group

For visceral pain, we found that oxycodone group has lower pain than sufentanil group at 2 h postoperatively (0.5(0,2.75) vs 3(2,4), *P* = 0.008 Fig. 3). When moving, the NRS was significantly lower in oxycodone group at 2 h (0.5(0,3) *vs* 3(2.25,4), *P* = 0.015) and 4 h (2(0,3) *vs* 3(0,4.75), *P* = 0.043) postoperatively.

One suffered postoperative respiratory depression and two suffered vertigo in the oxycodone group while one patient had recurrence of atrial fibrillation in the sufentanil group. Two patients suffered chest tightness in both groups respectively and were relieved after oxygen inhalation.

**Fig. 4:**
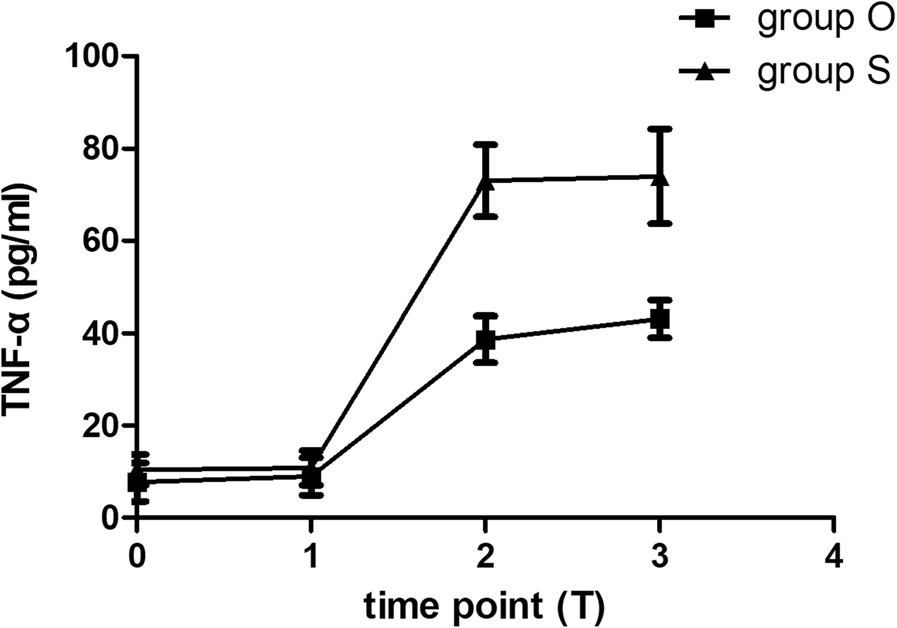
Serum Concentration of TNF-α. The square and the triangle is the mean and the transverse line is standard deviation. Group O = oxycodone group. Group S = sufentanil group

Serum TNF-α concentration in both groups gradually rose from preoperation to 24 h after surgery (Fig. 4). The comparison between the two groups showed the oxycodone group was statistically lower in TNF- α at 6 h (38.68 ± 10.49 vs 73.02 ± 16.27, *P*<0.001) and 24 h (43.12 ± 8.40 vs 74.00 ± 21.30, *P*<0.001) after surgery. For IL-6 and IL-10, no statistical significance could be found between the groups.

## Discussion

Preemptive analgesia is an effective strategy to provide adequate analgesia before patients are exposed to nociceptive stimulus. It decreases the physiologic reaction caused by pain and blocks the peripheral nociceptive stimulus transmition to the spinal cord, which inhibits hyperalgesia in central nervous system [18, 19]. Previous studies have shown that it could achieve better analgesic effect [20, 21]. Our study used equivalent dose of oxycodone and sufentanil as preemptive analgesia. Oxycodone takes effect about 2 min after injection, reaches peak level in serum in 3–5 min [22] and the duration of action is 3–4 h. Sufentanil takes effect in 3 min [9] and lasts for 0.5–1 h.

Previous study of Lenz et al. demonstrated that patients suffer most severe pain between 2 h and 6 h after surgery [23]. In our study, we found that visceral pain at 2 h and 4 h postoperatively were less with preemptive oxycodone. In addition, there was no difference on the incidence of adverse events between both groups. Preemptive oxycodone could be a good choice to reduce the hard to treat visceral pain in LC surgery.

Opioids is commonly used during surgery, but it is complicated by PONV, respiratory depression, hyperalgesia, opioid tolerance and anesthetic related mortality postoperatively [24–27]. These side effects are related to the activation of μ receptor by opioids. Sufentanil has strong affinity with μ receptor and it is easy to pass blood brain barrier with its highly fat solublity and achieves central analgesic effect. However, sufentanil is not the best choice in LC. The incision of LC is small and the visceral pain is the main component of intraoperative and postoperative pain. Peripheral κ opioid receptor has been suggested as a possible target for attenuating visceral pain [28]. Oxycodone has κ receptor agonist properties and is more potent in the treatment of visceral pain [29, 30]. It elevates the threshold for visceral pain stimulation [31] so that the pain signal is blocked in the peripheral and the input to central nervous system is then weakened [28]. In addition, the low fat solubility reduces the proportion of oxycodone passing through blood brain barrier so that the incidence of complications related to the μ receptor may be lower.

It is worth noting that there was no statistically significant difference on remifentanil dose between the two groups. This indicated that preemptive administration of oxycodone did not increase the dose of opioid used intraoperatively than preemptive sufentanil.

Whether analgesia is adequate could be reflected by the level of inflammatory factors during and after surgery. Previous studies suggested that TNF-α was the initial factor in systematic inflammatory reaction. It acts early and motivates the creation of other cytokines [32]. In addition, TNF-α also associates with pain and is involved in the formation and maintenance of hyperalgesia [33]. Song et al. demonstrated that TNF-α activation was critical in inflammatory visceral hyperalgesia [34]. These studies suggest that sufficient analgesia to reduce the TNF-α production is one of the most effective means to alleviate postoperative visceral pain. In our study, analysis of TNF-α showed that the concentrations at 6 h and 24 h after surgery in oxycodone group were significantly lower than the sufentanil group (*P* < 0.001). The results demonstrated that preemptive administration of oxycodone 0.1 mg/kg in LC could effectively suppress the release of TNF-α. According to the role of TNF-α in inflammatory reaction and pain management, we can infer that the systematic inflammatory syndrome in the oxycodone group is less than the sufentanil group. These findings are consistent with our observations of more reduction of visceral pain with preemptive oxycodone.

There are limitations in our study. First, some of the participants failed to follow-up at 24 h postoperatively because they have already been discharged and a serum specimen was not obtained. Second, the current study is not designed to evaluate hyperalgesia and incidence of chronic pain [33, 35]. Finally, duration of LC is short and surgeries with longer duration are needed for further observation on comparison between preemptive oxycodone and sufentanil administration on postoperative pain.

## Conclusions

For patients undergoing LC, preemptive oxycodone 0.1 mg/kg administration could effectively suppress visceral pain at 2 h and 4 h after surgery and inhibit the rising of serum TNF-α level at 6 h and 24 h after surgery without increasing postoperative complications. Preemptive oxycodone appears to be an effective strategy in dealing with visceral pain after LC.

## Data Availability

The datasets used and/or analysed during the current study are available from the corresponding author on reasonable request.

## Abbreviations

ASA: American Society of Anesthesiologists
BIS: Bispectral index
BP: Blood pressure
ERAS: Enhance recovery after surgery
HR: Heart rate
LC: Laparoscopic cholecystectomy
NRS: Numerical rating scale
PACU: Post anesthesia care unit
PONV: Postoperative nausea and vomiting
TCI: Target controlled infusion
TIVA: Total intravenous anesthesia
